# Supplementary material for: Ground penetrating radar observations of ancient large-scale deltaic structures in Jezero crater, Mars
Source: Sci Adv. 2026 Mar 18;12(12):eadz6095. doi: 10.1126/sciadv.adz6095 (PMC12998504; doi:10.1126/sciadv.adz6095)
Supplement: 20260318-1 [file sciadv.adz6095.v1.pdf]

PLANETARY SCIENCE

Ground penetrating radar observations of ancient large-scale deltaic structures in Jezero crater, Mars

Emily L. Cardarelli[1]*, Uni Árting[2], David A. Paige[1], Svein-Erik Hamran[3], Patrick Russell[1], Fredrik Andersson[4], Adrian Broz[5], Gwénaël Caravaca[6], Briony Horgan[5], Tor Berger[3], Sverre Brovoll[3]

Copyright © 2026 The Authors, some rights reserved; exclusive licensee American Association for the Advancement of Science. No claim to original U.S. Government Works. Distributed under a Creative Commons Attribution License 4.0 (CC BY).

The surface of Mars once hosted flowing liquid water and a warmer climate than today, with past water-rock interactions recorded by the carbonate deposits found on its surface. Here, we analyze the depositional setting of the Margin unit, a major magnesium-carbonate deposit near the fluvial inlet to Jezero crater using ground penetrating radar data collected by the Mars 2020 Perseverance rover Radar Imager for Mars Subsurface Experiment (RIMFAX) instrument. We report soundings from more than 35 m belowground, ~1.75 times deeper than other Jezero geologic units explored to date. We identify numerous subsurface features and submeter to hundred-meter scale layering across an ~6.1-km rover traverse. We infer that subsurface reflectors are consistent with buried fluvial features and deltaic foresets, which have experienced multiple erosional-depositional episodes. This work illuminates a well-preserved paleolandscape wherein a deltaic environment developed prior to the formation of the Jezero Western Delta, as early as the Noachian (~4.2 to 3.7 billion years ago).

INTRODUCTION

The surface of Mars hosts widespread evidence of ancient flowing water and paleolakes (*1*–*3*). Orbital and in situ spacecraft observations confirm the presence of aqueous alteration minerals on the Martian surface (*4*–*7*). Carbonate minerals are of particular interest because their formation and composition have implications for past interactions between Mars' carbon dioxide and water histories (*6*, *8*, *9*). The Jezero impact crater in the Nili Fossae region near Syrtis Major contains a paleolake basin with well-exposed fluvio-lacustrine delta deposits (*10*–*13*). The presence of secondary minerals such as phyllosilicates and magnesium-bearing carbonates (*4*, *14*, *15*) as well as primary minerals (i.e., olivine and pyroxene) indicates that the Martian surface has been aqueously altered (*4*, *16*).

The Margin unit is the largest exposed carbonate unit within the broader Jezero region and exhibits strong orbital carbonate and olivine signatures (*1*). It borders either side of the inlet valley to the Jezero crater lake and occupies the area between the crater rim and western fan deposits. The Margin unit's co-occurrence of carbonate and olivine lithologies coupled with an inferred history of fluvial regimes (*5*–*7*) were strong motivators to select Jezero crater as the landing site for the NASA Mars 2020 mission's Perseverance rover.

However, major uncertainties remain regarding how the marginal olivine-carbonate unit formed (*2*) and evolved (*8*) and whether it has preserved potential habitable conditions for ancient life (*2*, *4*, *9*–*12*). Here, we seek to understand the formational environment for the Margin unit of Jezero crater. The Margin unit has been proposed to have formed as a lacustrine beach deposit of paleolake Jezero (*2*), a Jezero crater inlet channel–based fluvial-deltaic deposit (*6*, *7*), a suite of altered pyroclastic flows (*13*) or ashfall-based deposits (*14*), or an altered igneous deposit (*9*, *15*) sourced by nearby regional volcanism (*16*).

[1]University of California, Los Angeles, Los Angeles, CA, USA. [2]Jarðfeingi, Faroese Geological Survey, Jóannesar Paturssonargøta 32-34, FO100, Tórshavn, Faroe Islands. [3]University of Oslo, Oslo, Norway. [4]Department of Earth and Planetary Sciences, ETH Zurich, Zurich, Switzerland. [5]Purdue University, West Lafayette, IN, USA. [6]Laboratoire Géosciences Environnement Toulouse, UMR 5563, CNRS, IRD, CNES, Université de Toulouse, 31400 Toulouse, France.
*Corresponding author. Email: ecardare@epss.ucla.edu

The Radar Imager for Mars Subsurface Experiment (RIMFAX) instrument aboard Perseverance provides a unique perspective into the shallow Martian subsurface, which in turn provides a potential window into habitable conditions on Mars (*17*–*19*). RIMFAX has acquired a continuous 6.1-km ground penetrating radar (GPR) image along the rover's Margin unit campaign path with soundings acquired every 10 cm. Radar soundings are presented from 78 traverses made by the Perseverance rover between September 2023 and February 2024, over 250 sols (i.e., mission days) (Fig. 1). Over the Margin unit campaign, Perseverance traversed 6.1 km and moved northwestward from the Upper Fan of Jezero crater, Mars across the Delta Blocky unit and onto the Margin unit. On the Margin unit, the rover traversed north and then westward across the Margin unit to the Crater Rim after visiting the Gnaraloo Bay region. The connection of Gnaraloo Bay to the Margin unit is not explored further in this work.

RESULTS

RIMFAX images the subsurface stratigraphy of the Margin unit to depths greater than 35 m (see Materials and Methods and Figs. 2 to 6). This unit is characterized by consistent deep soundings, reaching penetration depths that extend deeper than other units previously explored, including the Crater Floor (*18*, *20*), Western Fan (*19*), and Delta units (*21*). Strong radar returns (i.e., 400- to 550-ns two-way travel times) for all Margin unit drives are also reported (Figs. 2 to 6). The Margin unit differs markedly in its radar transparency from the Crater Floor units and Delta units documented in Jezero crater (*19*, *22*). RIMFAX two-way travel times are on the order of 500 ns compared to those observed in the Crater Floor and Delta units (~300 ns), from up to 1.75× greater (*19*, *22*) depths, from typically 15 m to more than 30 m belowground. The subsurface Margin unit medium is homogenous in that it is low loss, which enables deep penetration by RIMFAX for the entire length of the traverse examined (*22*, *23*). Velocities and subsequent depth belowground are estimated by fitting diffraction hyperbolas from the point scatterers present. These determined velocities range from 0.082 to

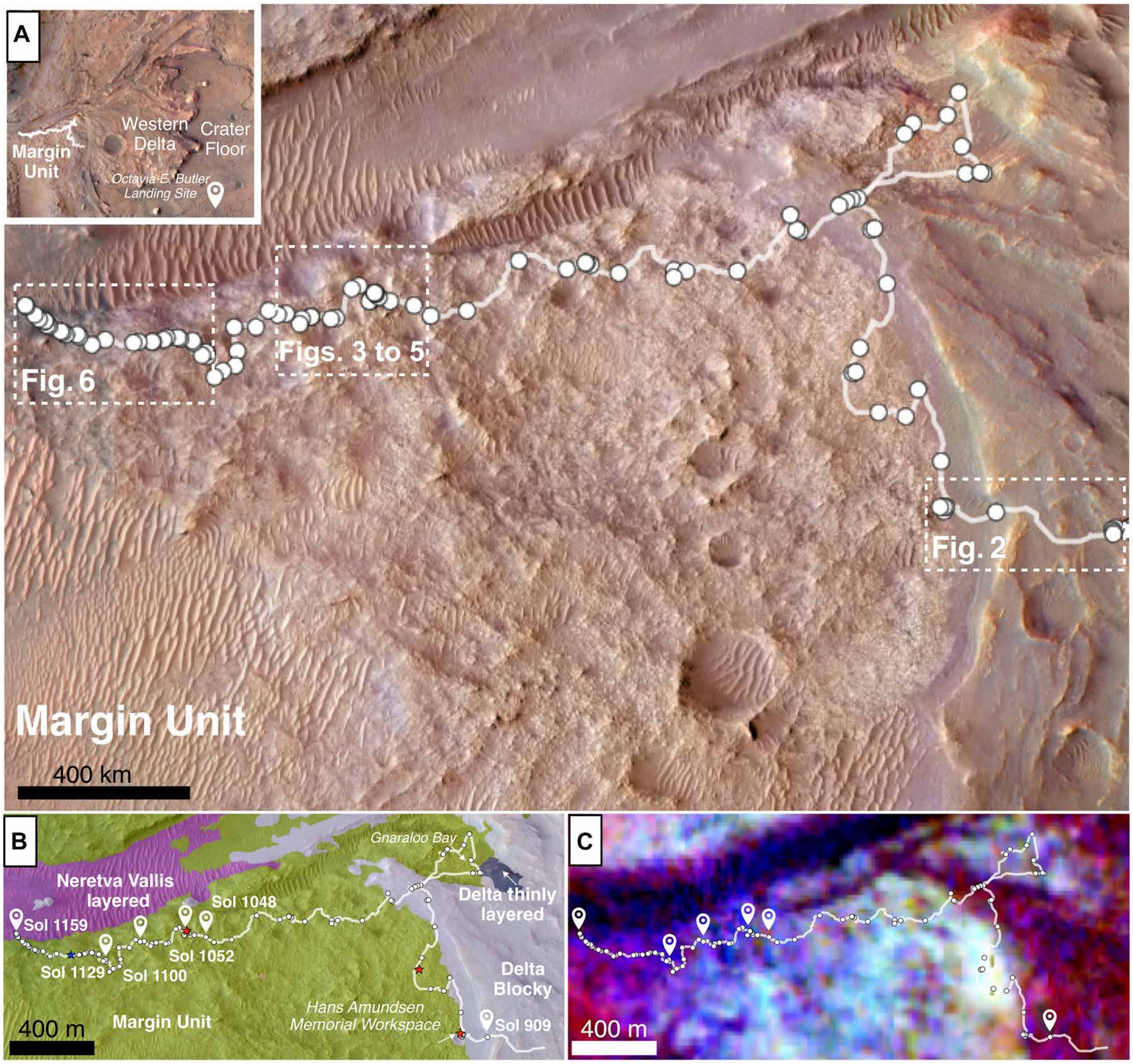

Fig. 1. Orbital context maps of the Jezero marginal region and its inferred spectral composition. (**A**) HiRISE mosaic of Jezero crater showing the rover path since landing (in white), with the Margin unit identified in a dashed white line inset. Red stars indicate where Margin unit abrasion patches and cores were acquired. A blue star indicates where only an abrasion patch was completed. (**B**) Prelanding geologic map of the Margin unit traverse (*3*) over Sols 909 to 1159 is presented with indicators and the specific sols highlighted in this work as white markers. (**C**) Compact Reconnaissance Imaging Spectrometer for Mars (CRISM) orbital spectroscopy map of the Margin unit traverse (*2*), wherein dark blue/turquoise corresponds to strong carbonate and weak olivine signatures, green indicates olivine poor with iron-bearing phases, red corresponds to olivine-dominated signatures, and yellow/white represents strong carbonate and strong olivine signatures.

0.188 m/ns, with a mean velocity of 0.126 m/ns (Fig. 7), which we assume for all radargrams. Prominent reflectors are visible from the ground surface to ~30 m belowground (Figs. 2 to 6). The upper boundary of the Margin unit was identified in the second half of Sol 909 drive, and the detection of the base of the Margin unit was identified in the traverse acquired on Sols 1129 to 1159. With these subsurface images acquired and the topographies of the traverses accounted for, we estimate that the Margin unit has a true thickness or actual vertical extent of at least 85 to 90 m (see the Supplementary Materials for additional topographic information).

Radar interpretation

RIMFAX GPR soundings detail strong and smooth radar reflectors that are laterally extensive to more than 100 m east-west, stretching beneath the Delta Blocky unit (Fig. 2). RIMFAX radargrams indicate that the Delta Blocky unit is characterized by numerous dark unorganized reflectors found at varying depths down to 12 m belowground on the Sol 909 traverse (Fig. 2; yellow unit in Fig. 2C). The signal scattering in the Blocky unit challenges the identification of subsurface structures in the Blocky unit or deeper Delta units from RIMFAX, while features down to the centimeter scale are visible more than 20 m belowground in the Margin unit (Fig. 2) due to lesser scattering and overall Margin unit material transparency. Multiple dark localized diffractions are found in the Margin unit as well as the Delta Blocky unit.

The Margin unit–Delta Blocky unit contact is present as a lateral layer dipping toward the Jezero crater basin at low angles (~5°) measured in the north-south direction and beginning a distance of ~30 m

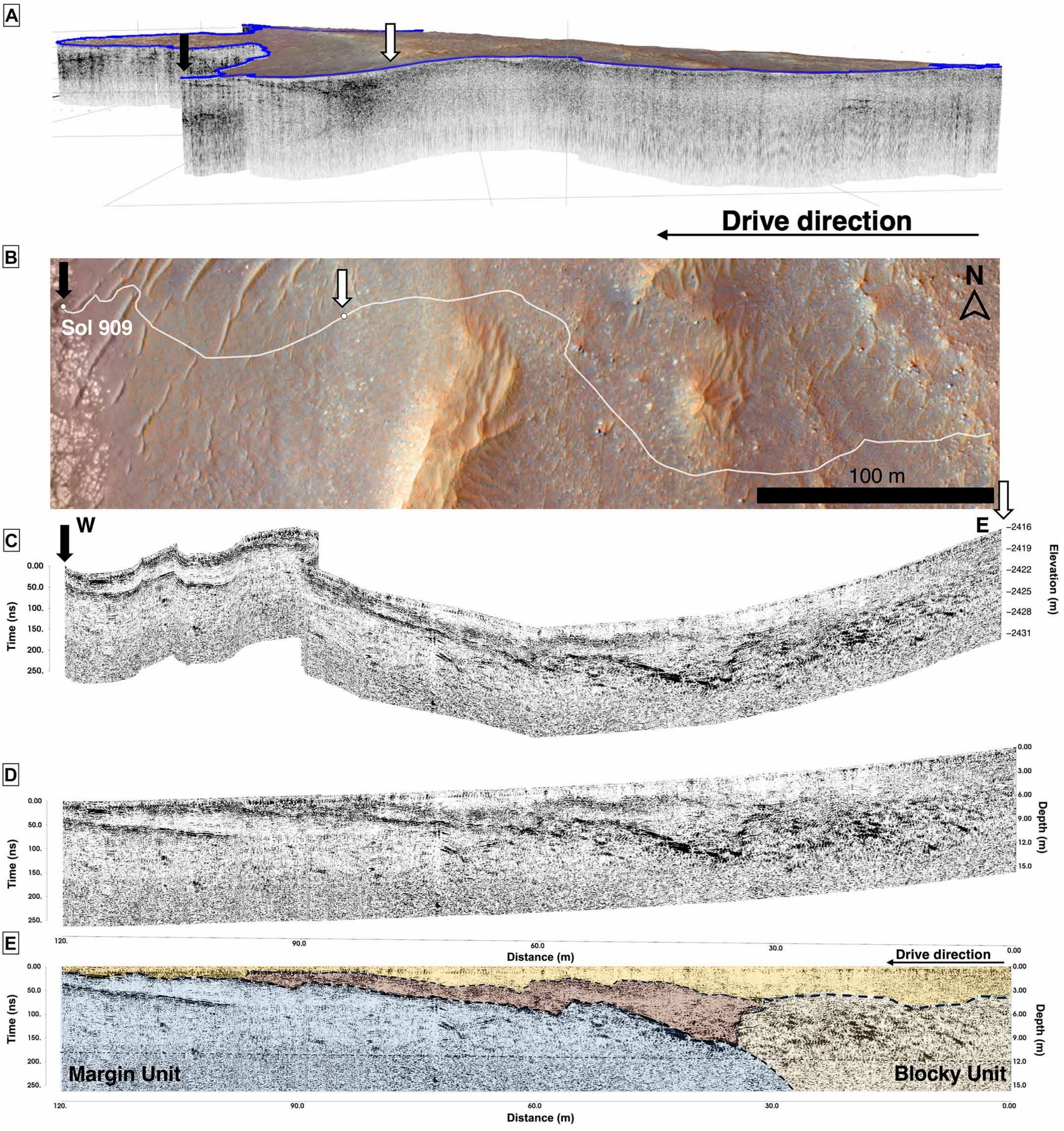

Fig. 2. Margin unit contact encountered on the Sol 909 traverse. (**A**) Three-dimensional (3D) side profile (2× vertical exaggeration) of the entire Sol 909 traverse encompassing a transition across the Delta units to the Delta Blocky unit (also dark in color), and to the layered Margin unit. (**B**) HiRISE mosaic of Sol 909 traverse with the white arrow indicating where the second half of the Sol 909 traverse begins and the black arrow at where the Sol 909 traverse ends. (**C**) RIMFAX radargram of the entire Sol 909 traverse shown with topography and acquired from the south, with the second half of the traverse extending from the middle white dot (white arrow highlights where the traverse begins) to the end of Sol 909 white dot (black arrow shows where the traverse ends). (**D** and **E**) Side view of the second half of the Sol 909 RIMFAX radargram with the Margin unit–Delta Blocky unit contact (dashed line), indicating that the Blocky unit onlaps the Margin unit, placing the Margin unit stratigraphically beneath the Blocky and Delta units. Multiple Margin unit layers are present, and similarities are apparent between the Blocky unit found at the surface as well as buried at depth. (C) to (E) are at 1× vertical exaggeration.

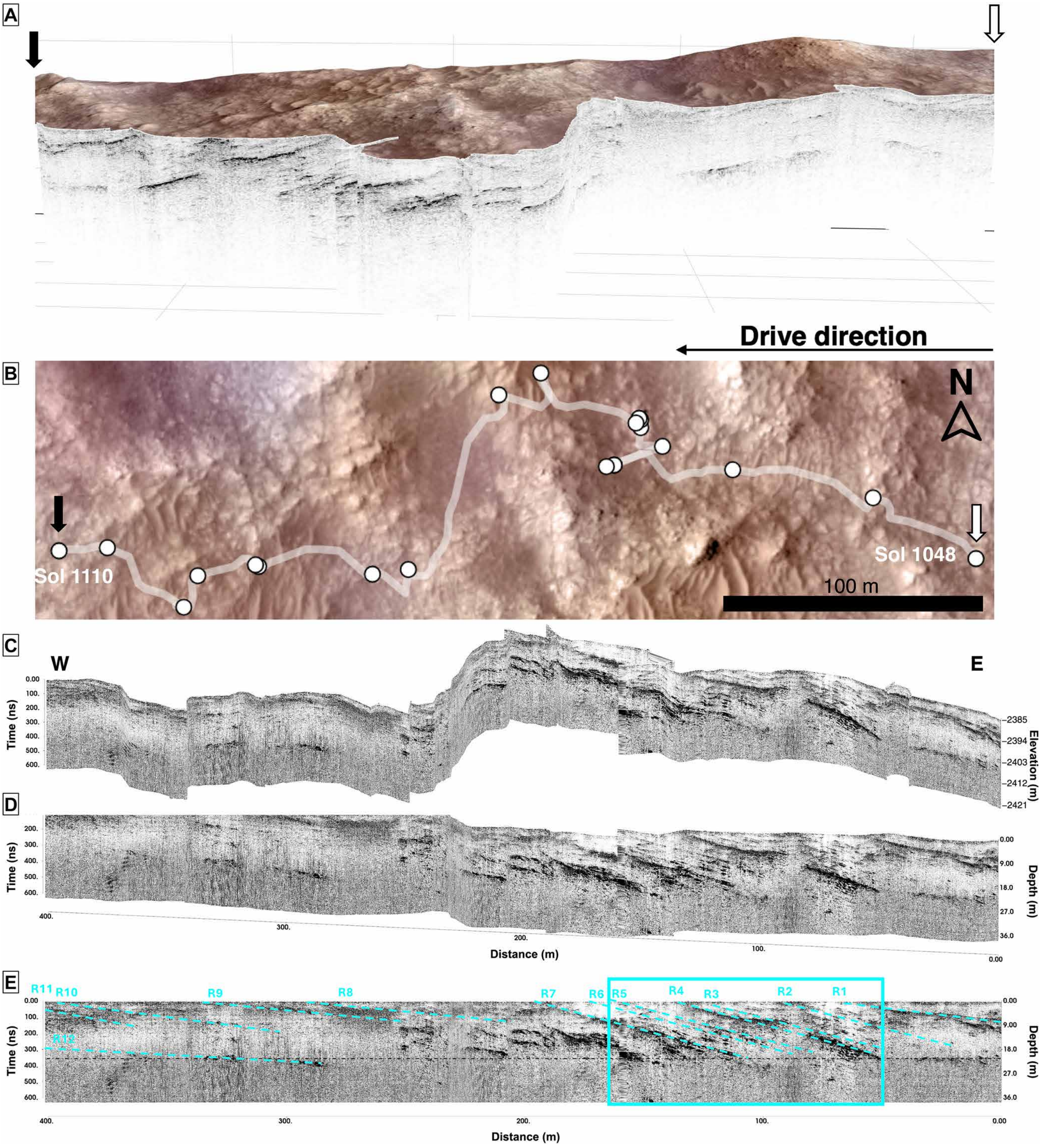

Fig. 3. Regional dipping beds in the western Margin unit. (**A**) 3D of the side view (2× vertical exaggeration) of the RIMFAX radargrams over Sols 1048 to 1110 integrated with the HiRISE topography. (**B**) Top-down view of the Sol 1048 to Sol 1110 traverse visible within the HiRISE map. (**C** and **D**) 3D projection of the drive traverse. (**E**) The radargrams acquired over Sols 1048 to 1110 (C to E) indicate regional dipping beds [cyan dashed annotations on (E)] that are laterally continuous over visible and characterized over the traverse [(A) and (B)]. The identified layers (R1 to R12) dip toward Jezero crater at 3° to 15° determined from a migrated profile, and the cyan box outlines the details found in Fig. 4. (C) to (E) are at a 1× vertical exaggeration. N, North; W, West; E, East.

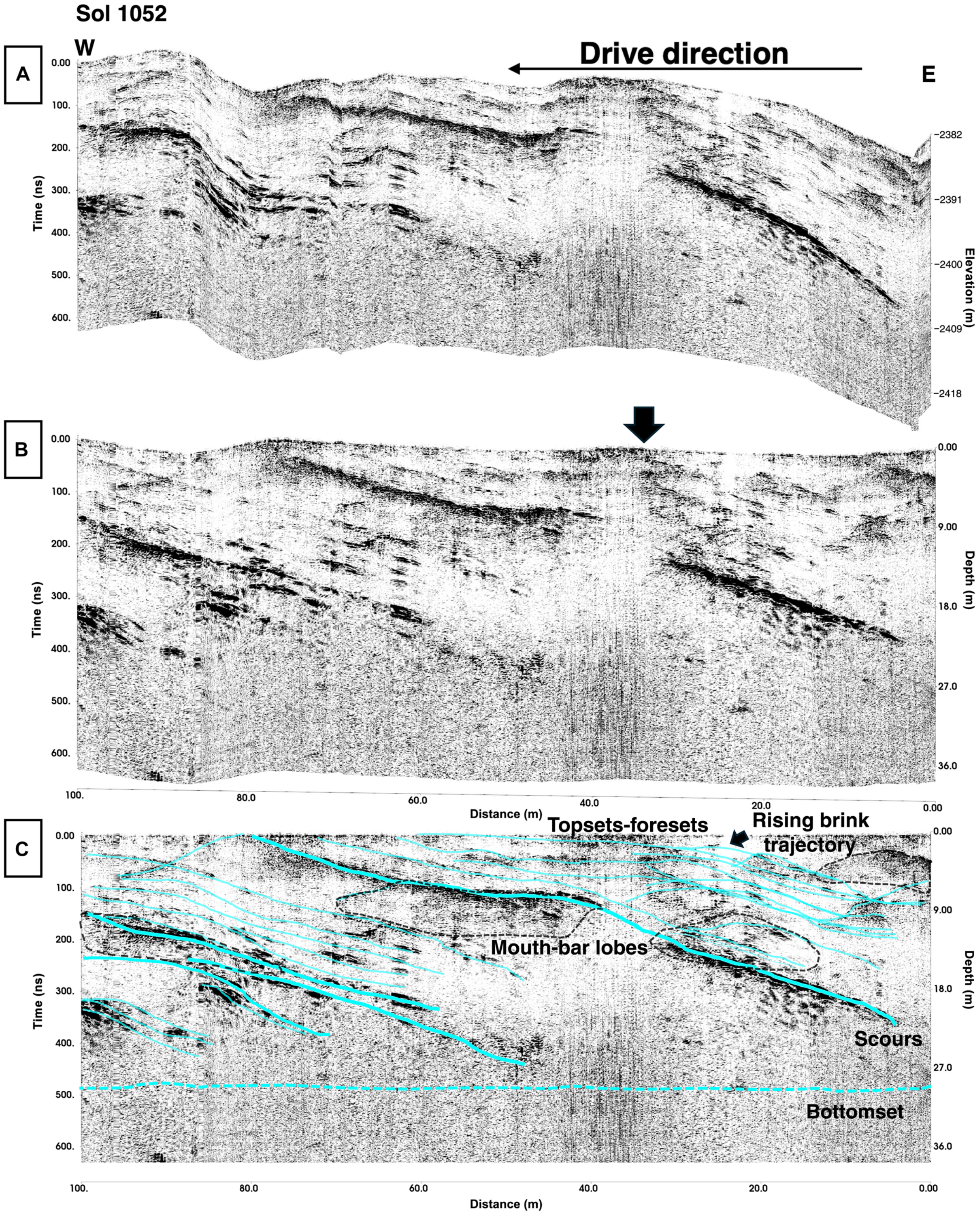

Fig. 4. Margin unit traverses highlighting western Margin unit sedimentary structures observed on Sol 1052. (**A** and **B**) 3D visualization of Sol 1052 traverse indicating the geometry of layers present. (**C**) Side view of a 3D visualization for the Sol 1052 traverse, with annotations highlighting the presence of clinoforms, Cr, Bs, scours, and mouth-bar lobes. Ol and multiple rising brink trajectories are present. All radargrams have an assumed velocity of 0.126 m/ns.

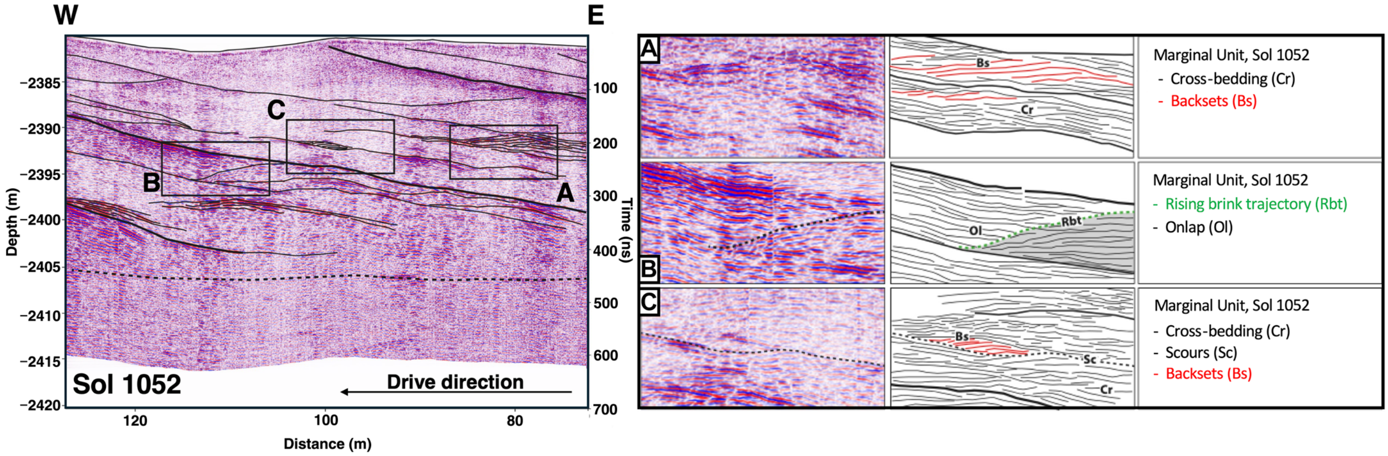

Fig. 5. Margin unit traverses highlighting western Margin sedimentary structures observed on Sol 1052 with fluvial features annotated. Central reflectors are enlarged and detailed further (**A** to **C**).

after the start of the Sol 909 traverse at a depth of −2431 m and extending to the end of the Sol 909 traverse, rising from 15 m belowground to less than 1 m belowground (Fig. 2). Within the Blocky unit, there is also an undulating surface that consistently has darker shading levels and multiple meter-scale reflectors at ~9-m depth and 20 m from the start of the traverse. Adjacent to the Blocky unit, there is a layer of medium-intensity reflectivity with irregular, sinuous boundaries (Fig. 2, light brown layer) that follow the Margin unit boundaries below it. These dipping reflectors appear overtopped by surficial layers that are distinct radar facies to the Margin unit (Fig. 2E, yellow and brown layers).

The Margin unit hosts multiple Margin unit layers (light blue unit with layers, Fig. 2C) characterized by high reflectivity. These high-reflectivity layers also have intercalated low-reflectivity regions between them that appear dark in color in radargrams (Fig. 2C). These extensive layered features are also widespread in the western traverses of the Margin unit (Figs. 3 to 6) and are offset, yet parallel, to the Sol 909 traverse. RIMFAX observed strong subsurface reflectors (R1 to R12) on the Sol 1048 to Sol 1100 traverses, which totaled a distance of 326 m (Fig. 3). While the dip angles range from 3° to 15°, these basinward dipping beds appear to be repeatedly spaced (Fig. 3) and a characteristic feature of the Margin unit (figs. S1 and S2). These reflectors are laterally continuous over several hundred meters with a consistent dip direction (Figs. 3 to 6) toward the basin and fan/delta deposits of Jezero crater that are lower in elevation (*19*, *21*). These extensive repeating reflectors also continue beyond Sol 1100 and are observed as far as Sol 1159, with individual reflectors extending laterally to ~200 m (Fig. 6).

RIMFAX also identifies numerous subsurface reflectors that are present with complex architectural elements on the Sol 1052 and Sol 1055 drives (119.5 m total) (Figs. 3 to 5 and fig. S2). At first order, three major reflectors are uniformly spaced (~20 to 30 m) and laterally continuous (extending to ~100 m in length). These features dip toward the basin at 3° to 15° with the steepest reflectors being R2 to R7 (Fig. 3), and at the base of these first-order features, a lower horizon may be present at 500 ns (Figs. 4 and 5). Second-order features include a lensoidal distribution of internal reflectors (Figs. 4C and 5) wherein complex, oblique truncated layers are on the order of 10 to 20 cm each and found in packages that are ~1- to 2-m thick.

Geologic interpretation

The Margin unit contains subsurface structures at hundred-meter to submeter scales (Figs. 2 to 6) that are comparable to those previously observed in surficial exposures in the Jezero crater Delta Front (*21*) and on similar scales in analog environments on Earth (*24*). Internal multiscale structural details with identifiable features are visible at a decameter scale down to the tens of centimeters scale on Sols 1048 to 1110 (Fig. 3) and Sol 1052 (Figs. 4 and 5 and fig. S1). Detailed interpretations of submeter-scale reflectors reveal various sedimentary structures such as cross-bedding (Cr), onlap relationships (Ol), and backsets (Bs) (Fig. 5, A to C). There may also be an inferred rising brink trajectory moving through the features diagonally (Figs. 4 and 5). Surface expressions of the RIMFAX layers are present on the eastern side of the Margin unit, although erosional events have disrupted the surface so they are often not obvious as outcrops. However, subsurface RIMFAX layers are visible at the surface in the western traverses of the Margin and are particularly well exposed around the Bunsen Peak area (Sols 1052 to 1055, figs. S1 and S2). These layers appear as eroded, laterally extensive layers (cyan traces, figs. S1 and S2) dipping toward the basin, which are best observed from the rover looking toward the basin at the backside of the hummocks we traversed between (cyan traces, fig. S1).

Multiple inferred fluvial events are preserved in the sections examined (Figs. 3 to 6). We report complex architectural elements and internal reflectors encountered on the Sol 1048 to Sol 1100 drives (Figs. 4 to 6), which are consistent with both Gilbert deltaic features (*25*–*27*) and fluvial features (*24*, *28*–*30*). Within this traverse extent, structures are also visible in localized areas that co-occur with some regional reflectors. We interpret the major reflectors detailed at first and second orders in the Sol 1052 and Sol 1055 traverses (Figs. 3 to 6) to be multiple visible clinoforms that are parallel and horizontally continuous and span roughly 80 m in length. The clinoform features transition from topsets to foresets at ~25 to 35 m. The rollover point within these clinoforms is visible as is its movement to the upper right as well as the movement of the rollover point on potential foresets (Fig. 4). A lower horizon found below the topsets and foresets at ~500 ns is interpreted to be a bottomset, as it is laterally extensive and the facies are consistent across the traverse. These first-order reflectors observed may be consistent with foresets that

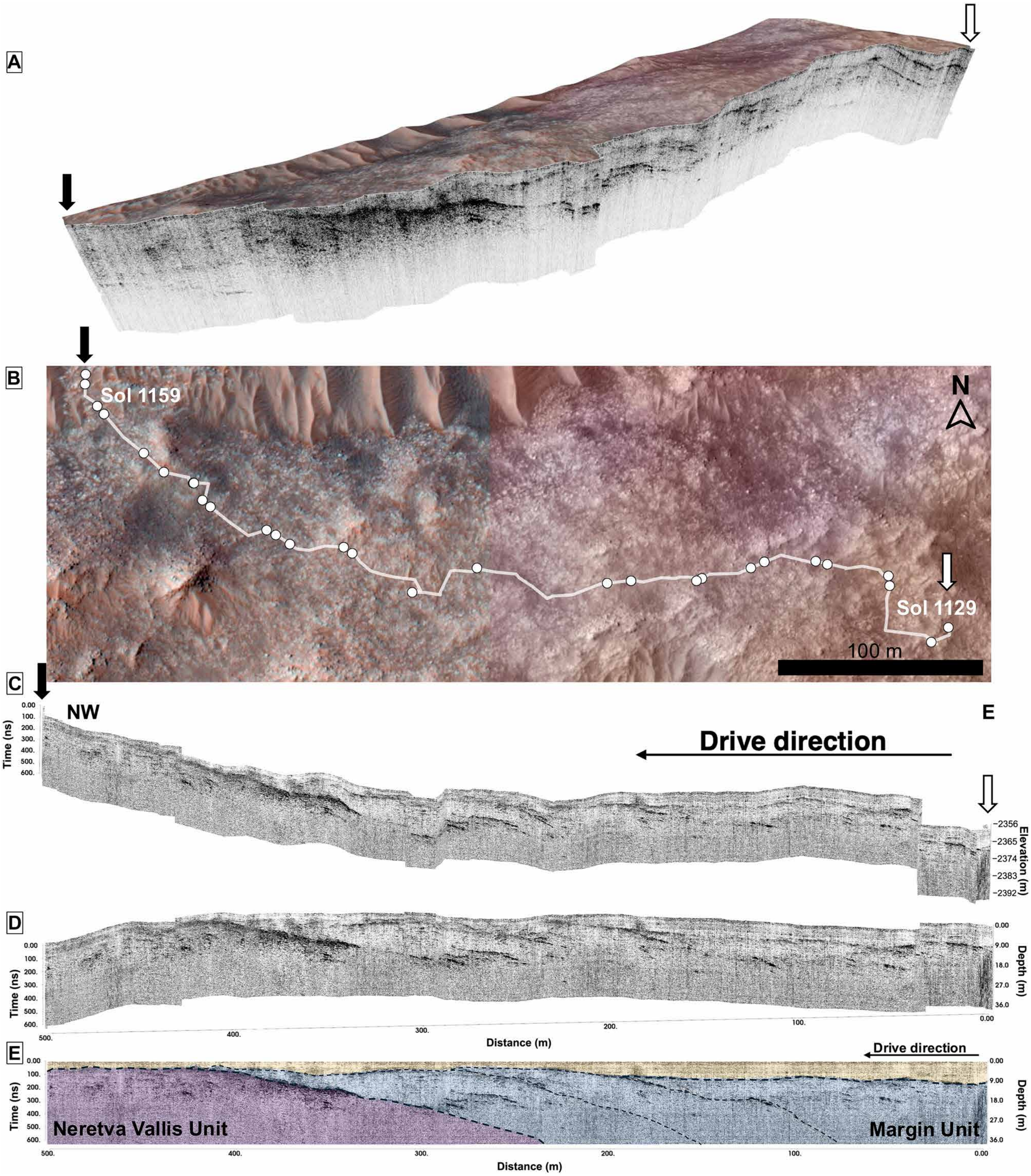

Fig. 6. RIMFAX radargrams from Sols 1129 to 1159 driving westward. (**A** to **C**) Viewed from the south, traverse distance from the end of the drive (in meters) is shown relative to depth [in meters (m)] and travel time [in nanoseconds (n)]. (**D** and **E**) The drive from Sols 1129 to 1159 viewed from the side profile hosts multiple uneven, bumpy surfaces that are both at the surface as well as at 300 ns, which are inferred to be erosional surfaces. Multiple laterally extensive layers are present within the Margin unit (blue units) and a Neretva Vallis unit at depth (purple). Over all units, a surficial layer as well as an onlapping erosional surface to these layers (bright blue) is present. Some Margin unit layers appear to come close to the surface of the unit, although all the Margin unit layers appear to be overlain by a shallow layer of regolith (light yellow). The potential basal contact of the Margin unit is also visible as the strong reflector at ~300 m in the traverse (purple, farthest left). NW, Northwest.

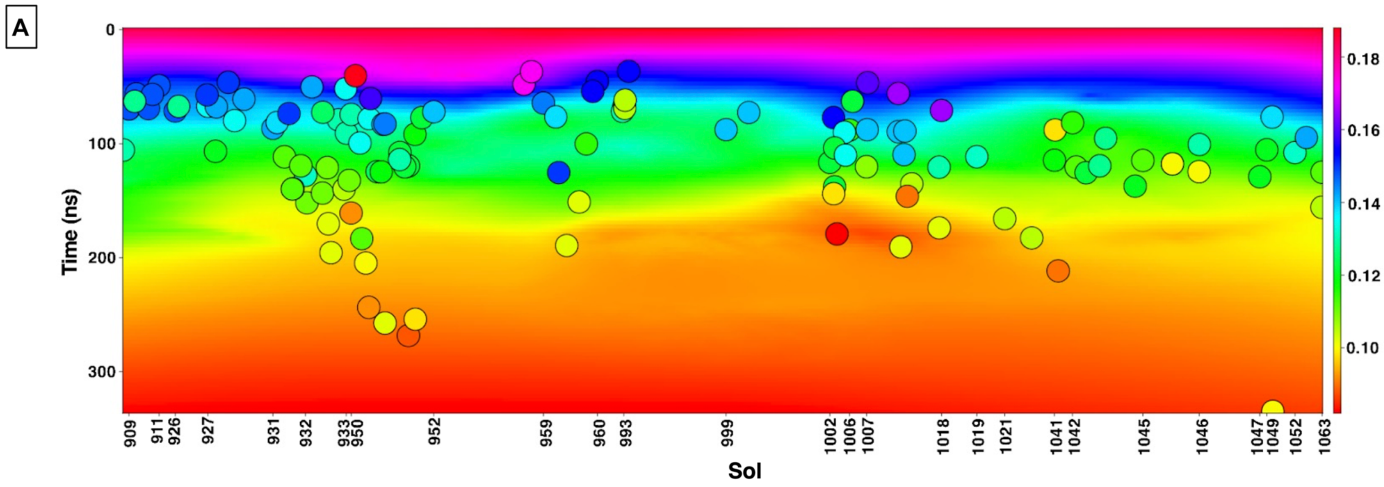

B

Subsurface material	Number of hyperbolas	Mean dielectric permittivity ε′	Mean diffractor depth (m)	Mean velocity (m/ns)	Inferred mean density ρ (g cm^{-3}) using ε′ = 2.0^{ρ}
**Margin Unit**	126	6.14 ± 2.08	15.49	0.126 ± 0.02	2.62
**Lower Delta sediments**	11	7.18 ± 1.32	3.12	0.113 ± 0.013	2.84
**Crater Floor**	150	8.9 ± 3.2	1.9	0.10 ± 0.02	3.1

Fig. 7. Margin unit velocity estimates and subsurface dielectric properties. (**A**) Velocity estimates from parabolas in the subsurface are shown relative to sol (*x* axis) and time (in nanoseconds; *y* axis). The velocity estimates of each parabola are colored by their velocity values and shown relative to the sol they were determined on and time. The color bar is in meters per nanosecond (m/ns) and corresponds to both the points and figure. Velocities range from 0.082 to 0.188 m/ns with a mean and median velocity value of 0.126 m/ns. (**B**) Margin unit, Delta, and Crater Floor subsurface dielectric properties.

have undergone surficial erosion and later deposition (Figs. 5 and 6), similar in size to features observed with the Delta unit of the Jezero crater (*31*) and sedimentary units in the southern Utopia Planitia region (*32*). These first-order reflectors resembling foresets could also be consistent with shoreline structures or strand plains; however, the presence of multiple and laterally successive rollover points suggests that the features are deltaic foresets over shoreline features.

At the contact of the Margin unit and the Upper Fan, the Sol 909 traverse indicates that the Upper Fan units of the Jezero crater delta onlap the Margin unit (Fig. 2). The Margin unit underlies the Upper Fan units and thereby predates the Jezero Western Fan visible from orbit. The Delta Blocky unit appears to be a superficial unit that has been emplaced after the Margin unit and other Delta units (Fig. 2, A to C). A contact between the Margin unit and the Delta units where the Delta units onlap the Margin unit could exist, although the Blocky unit radar facies may obscure deeper detections of other units beyond its dense boulder-containing layer. Both the regionally extensive layers as well as the dense, fine-scale (subcentimeter scale) layered features of the Margin unit are underlying the Delta Blocky unit (Figs. 2 and 3). These equally spaced layered reflectors appear to have experienced multiple erosive and depositional events, resulting in additional horizons and uneven surfaces overlying them (Sol 909, Fig. 2; Sols 1129 to 1159, Fig. 6). These structures suggest deposition under consistent energy conditions evidenced by laterally extensive horizontal layers, which are also spaced at equal intervals (Fig. 3). By contrast, the steepened reflectors (R2 to R7, Fig. 3) indicate variable energy conditions during deposition.

RIMFAX can constrain the lowest elevation for the Margin unit at −2431 m through its initial detection made on Sol 909, traversing across the Margin unit–Delta Blocky unit (Fig. 2) contact before reaching the Hans Amundsen Memorial Workspace. Within this workspace, the orbital High Resolution Imaging Science Experiment (HiRISE) (*33*) would approximate the lowest surface expression of the Margin unit to be −2422 m (Fig. 1) and 9 m higher than the contact at depth revealed by RIMFAX. Overall, steep surface topography is observed in the northwestern and western localities of the Margin unit (Fig. 3).

Elevations increased as the rover traverse proceeded in the northern and western directions, ascending ~55 m in elevation throughout the drives examined in this campaign. The elevations summarized over the radargrams of the unit (figs. S1 and S2) indicate the stratigraphic thickness of the Margin unit to be ~85 m. The upper Margin unit contact with the lower Delta Blocky unit is observed on the Sol 909 traverse (Fig. 2) at ~14 m belowground at −2431 m, and the highest basal contact of the Margin unit with the underlying Neretva Vallis unit is observed as the rover approached the rim of Neretva Vallis at −2350 m (Fig. 6).

DISCUSSION

The detailed three-dimensional (3D) architecture and geometry of the buried features as well as comparisons of structure and morphology with other environments previously observed by RIMFAX together point toward a sedimentary origin for the Margin unit. Our analyses suggest a subaqueous Gilbert-type deltaic formation as the most likely depositional environment for the Margin unit. To support this interpretation, we present a broader orientation of the Margin unit relative to the Jezero crater and highlight how surficial features in the Margin unit are linked to our subsurface observations. Last, we evaluate alternative formational hypotheses and summarize how our implications affect our understanding of water within Jezero crater.

Past orbital studies and in situ studies show that the Margin unit deposits are centered on a paleochannel that was likely the original inlet valley that incised the rim wall (*1*, *2*, *8*) of Jezero crater (*2*). Mars terrestrial environments, such as the martian Saheki crater alluvial fan, have developed distributary networks of fluvial channels with fan thicknesses of 850 m (*34*), whereas Earth-analog alluvial environments have developed decameter-sized alluvial deposits (*24*, *28*, *29*). Thus, the architecture of the Margin unit supports the presence of a sizable in-flow channel or inlet valley. In addition, angled layers truncate along a bottomset (Figs. 3 to 5), which could be interpreted as the base of the Margin unit or alternatively an undetermined layer that is relatively shallow in thickness. A transition between Margin unit layered facies and underlying layers is also present on the Sol 1129 to Sol 1159 traverses (Fig. 6), which could represent the basal contact of the Margin unit.

Mars Orbiter Camera imagery indicated that the Margin unit surface is composed of layered ridges of sediment that cross-cut each other, which were interpreted to be coarse channel bed material deposited as the valley entered the crater (*7*). Our work indicates that these ridges are linked to subsurface structures (fig. S2) and that these depositional events occurred with regularity. However, steepened foresets (R2 to R7, Fig. 3) reflect specific depositional events that occurred under high-energy depositional events and/or rapid delta progradation conditions. Low-angle foresets before (R1, Fig. 3) and after (R8 to R12, Fig. 3) these events thereby suggest that the Margin unit was a more stable depositional environment or had lower sediment supply conditions before as well as after these events. In southern Utopia Planitia, the Zhurong rover team reported similar reflector orientations and inferred that they were due to coastal sediments depositing into a large water body (*32*). Our work presents a similar sized depositional body and reflector angles in the range Zhurong reported (3° to 20°) (*32*), with the presence of a nearby suspected inlet channel (*35*). Given the widespread distribution of the laterally extensive reflectors we have detected, which are inferred to be due to episodic subaqueous depositional events, and these recent Zhurong findings, might these observations insinuate that broad-scale ancient deltaic deposition occurred in the Noachian or a regional ancient Martian ocean was present?

Paleolandscape relationships revealed through subsurface stratigraphic profiles

Potential formation environments for the structures observed could include (i) a braided river system to an alluvial fan to a lake (*36*), (ii) a meandering river system with an alluvial fan (*37*), (iii) a nontrenched fan (*38*), (iv) a fan head entrenchment in a proximal fan (*38*), (v) nested fans (*37*), or (vi) a climatically controlled fan (i.e., fan head entrenched over various potential climate regimes) (*34*). A nontrenched fan environment may be present as beds appearing stacked and dipping away from the water/material source when a vertical transect through a fan and multiple alluvial fans are present. The features may also appear to be lensoidal or hummocky deposits if oriented perpendicular to the fans. Regional observations of extensive lateral beds with consistent dips toward the channel and hummocks or lensoidal features of varying sizes and internal complexity are a record of the active formational environments. A fan head entrenchment in proximal fan environment may appear as areas where there are short beds in parallel due to the dendritic fingers of the fan (*39*), and this would appear as a vertical transect through a fan and/or multiple alluvial fans. This interpretation could be a plausible hypothesis also for the wide truncated beds present, which top the fine-scale features reported (Sol 1052, Figs. 4 and 5). In comparison, an environment of nested (e.g., superimposed) fans could also appear as a series of diagonal erosional surfaces and uneven or stepped beds. This might be due to an erosive channel flowing through the landscape as a vertical or diagonal transect among the multiple various depositing fans. Potential examples may be where erosional boundaries/bumpy contacts/scours or where edges of clinoforms might be present. Last, an environment representing a climatically controlled fan might appear as a vertical transect through a stabilized fan surface where multiple beds would be present in cross section and be layered (*34*, *37*). These layers likely represent the stabilized fan surfaces through time, with the deeper layers indicating greater maturity. This setting may be observed in the radargrams, which show regionally extensive lateral beds dipping at consistent angles toward the paleo-inlet channel.

The presence of various types of radar reflection signatures over a large spatial extent and elevational gradient enables an investigation of the factors affecting sedimentation, preservation, and lateral and vertical cross-sectional profile development. These include the geomorphic structures that may have subsequently formed (*25*, *38*). Geologic relationships and crater retention ages from orbital images have suggested that the Margin unit could be anywhere from 3 to 4 billion years in age (*2*, *8*, *9*). RIMFAX observations indicate that the Margin unit underlies the boulder-rich units as well as the Delta Fan units (*19*) and thereby possibly represents the stratigraphically oldest unit examined in the broader Jezero delta area.

The Margin unit has characteristics that indicate that its formation is consistent across a regional scale of the western Jezero crater. Differences exist within local areas in the subsurface of the eastern Margin unit as compared to the structures in the western Margin unit. However, this work presents multiple potential settings (alluvial fan, floodplain, and braided/meandering river) that are recorded in the subsurface, although further narrowing down the exact depositional timing is challenging with the current dataset. All possibilities

envisaged here are linked more broadly to a fluvio-deltaic setting (Figs. 5, 6, and 8).

Delta environments can become divided into subenvironments due to factors such as topography and slope gradient as well as depositional/erosional processes from differing flow regimes (i.e., Gilbert delta progradation to hyperpycnal delta progradation) (*40*). These result in distinct facies types and subsurface structures (*29*). A fluctuating endorheic deltaic-fluvial system that retained water was likely present, implying that the complex surface topography retained water and potentially carbonated the lithology. Earth analog sedimentary fans are also characterized by highly complex paleosurfaces, with local differences in fan behavior, making inter-regional comparison of paleosurface stratigraphies challenging. Areas relatively close to one another but with different geology, relief, and microclimates may not only display different depositional styles but also respond in different ways to changes in climate (*41*). For example, short periods of stability could have occurred on abandoned fan segments as there are few deep cut-and-fill sequences observed.

The identification of compelling fluvial features (e.g., topsets, foresets, and mouth-bar deposits; Fig. 3) is consistent with a sedimentary fluvial interpretation for the Margin unit. This interpretation includes a preserved past alluvial fan environment demonstrated by features that include hummocks in the cross section as well as lateral observations of tilted beds. These subsurface observations of Jezero crater's Margin unit suggest sedimentary facies and a potential fluvio-deltaic setting, which may transition to an alluvial-endorheic basin setting. These potential formation environments are difficult to differentiate on Earth as well as on Mars. However, they all hold in common that they suggest and record substantial water-rock interactions as well as alluvial structure building, which support the preservation of sedimentary fabrics and water-rock interactions at unprecedented depths revealed in the Margin unit.

This work also may have implications for the preservation of potential biosignatures and habitability in the subsurface of Jezero crater. Grain size, as well as lithology at depth, can only be moderately estimated from radar travel times and return signal, although these identified fine-scale internal structures could preserve mineral compositions and geochemical conditions of past water-related events and may have once provided past habitable conditions. Internal structure in subsurface alluvial environments on Earth supports microbial diversification and promotes geochemical niche development (*42*, *43*).

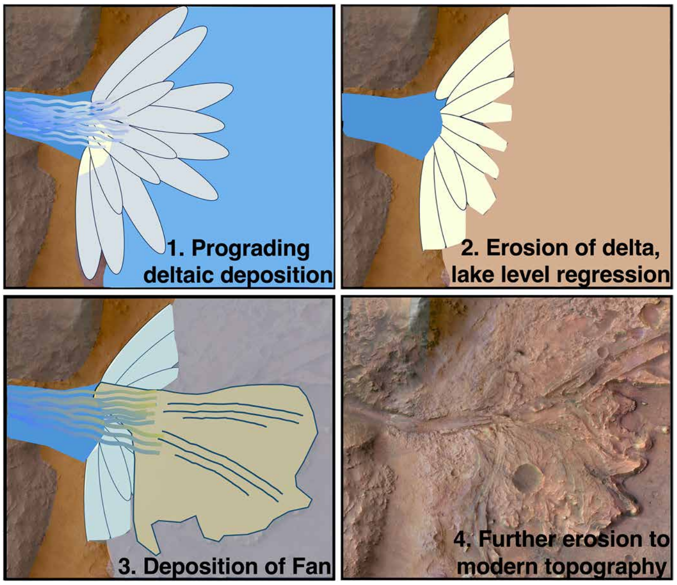

Fig. 8. Reconstruction of the depositional and erosional history of the Margin unit in Jezero crater based on radar facies from RIMFAX traverses. (1) Radar reflectors suggest that there was an ancient large-scale Gilbert delta that deposited a series of large-scale lobes through a single channel or smaller subchannels into Lake Jezero and produced the Margin unit. (2) These delta deposits underwent extensive erosion over time that reduced the deposits and textures found on the surface. (3) Following the erosion of these ancient deltaic units, the Jezero Delta and Fan units were deposited subaqueously and overlie the Margin unit. (4) Ongoing erosional activity has altered the subsurface as well as the surficial expressions of the geologic units present producing the modern topography visible in HiRISE images today.

Alternative formational hypotheses

Alternative hypotheses were considered to explore whether the observed radar reflections could be a result of other depositional or alteration processes. It has been previously suggested that the Margin unit is an outcrop of a broader regional olivine-carbonate bearing unit in the greater Nili Fossae area formed (i) by igneous processes such as (a) extrusive volcanism activity (including pyroclastic/volcaniclastic events and volcanic ashfall) due to similar spectral signatures and geomorphic features (*1*, *4*, *9*, *14*, *15*) or (b) igneous intrusions, (ii) as alteration by-products of plausibly igneous material, or (iii) by sedimentary processes other than fluvial-deltaic such as (a) lakeshore deposits or (b) glacially driven processes.

Extrusive igneous

The Margin unit shares consistent olivine as well as carbonate signatures with areas outside of Jezero and in the greater Nili Fossae area (*4*, *9*, *15*, *44*), which is hypothesized to have had active volcanism for the past billion or more years. A hypothesis for this widespread regional consistency of spectral signatures is that distal extrusive volcanic processes at some point in time generated an ashfall (*14*) and/or pyroclastic material (*13*) that was compacted and aqueously altered over time. Following this hypothesis, we would expect to see pyroclastic layering through multiple debris flows and/or draping ashfall deposits (*13*–*15*). If the Margin unit was produced through extrusive volcanism, we would also anticipate that the density of the material would be heterogeneous on the surface over a large-scale traverse due to variable cooling rates, rheological properties, and flow composition. We would also expect that the subsurface densities would be heterogeneous with depth relative to how the bodies cooled, with greater densities in the center of the bodies or layers relative to the edges of the units (*45*, *46*). However, RIMFAX imaged homogeneous and consistent features across the Margin unit region as well as a paleofluvial channel bisecting the Margin unit (Fig. 6), unlike a traverse-dependent and heterogeneous subsurface that would be expected from an extrusive igneous origin. We also see an overall thickness estimate of the Margin unit of less than 100 m as well as the bottom of the Margin unit, which would be unexpected for a proposed massive extrusive body of volcanic origin sourcing the lithologies observed. With the identification of mouth-bar lobes, backsets, cross-bedding features, and clinoforms in the Sol 1048 to Sol 1110 region coupled with the presence of repeated layering at uniform spacing, an extrusive igneous formation hypothesis is therefore not the preferred interpretation for the Margin unit of Jezero crater. The formation of the aforementioned features repeatedly formed and found together is seemingly unlikely due to extrusive volcanism activity.

Intrusive igneous

The Séítah unit of the Crater Floor shares an olivine-bearing orbital spectral signature with the Margin unit (*4*, *9*, *14*). In situ geochemical and geophysical data suggest that the Séítah unit of the Crater Floor is an olivine-cumulate with layers (*22, 47*). Following the hypothesis that the Margin unit is connected regionally to other nearby olivine-bearing deposits, the Margin unit may also be an intrusive igneous body like the layered Séítah unit of the Crater Floor and the result of broader intrusive volcanism processes that generated an extensive layered cumulate now exposed in the Margin unit as well as in the Séítah unit of Jezero crater. Under this scenario and based on layered igneous intrusions on Earth, we would expect to see the presence of an intrusive complex in Jezero crater on the order of one to several kilometers in thickness (*45*, *48*–*50*) as well as eroded legacy sediments. Layered features found in such deposits could develop on the decameter and submeter scales through multiple igneous intrusions (*48*). However, an igneous intrusive hypothesis is not the preferred interpretation for the Margin unit because we observe geometries of laterally extensive repeating units with uniform spacing and with similar thicknesses. These repeating angled layers truncate along a bottomset or basal layer, and we detected the local bottom of the Margin unit at ~35-m depth below the surface (Sol 1052, Fig. 4), the unit basal contact (Sols 1129 to 1159, Fig. 6) and consistent deep penetration depth across the unit. These observations and the lack of eroded igneous sediments ponded in the local vicinity lead us to favor the interpretation that the Margin unit has an origin other than as an intrusive igneous body (*18*, *24*, *46*, *48*).

Past orbital observations have contended that the greater Nili Fossae region has undergone widespread liquid water activity and a multitude of alteration processes (*15*, *35*, *51*). If the Margin unit formed from the aqueous alteration of an aforementioned igneous cumulate deposit or the aqueous alteration of an ignimbrite, then we would expect the deposit's alteration to be anisotropic and variable in its extent. In Séítah, we observed lithologies that were heterogeneously and partially carbonated, although the igneous Crater Floor did not exhibit evidence of large-scale carbonation (*47*, *52*, *53*). However, we observe homogeneous, uniform large-scale structures on the Margin unit. These observations contrast our expectations of in situ carbonate and may suggest a large-scale depositional deltaic environment that sourced multiple lobes varying in depositional orientation and extent, evolving vertically, horizontally, and laterally.

Lakeshore deposit

If the Margin unit was the result of lakeshore or shoreline deposits in paleolake Jezero (*2*), then we would expect the GPR reflections observed to be low-angle, horizontal reflectors that extend up to the decameter scale (*54*, *55*) and be consistent with shoreline deposits and/or beach ridges that have developed. However, we observe prograding aggradational clinoforms seen on Sol 1052 (Figs. 4 and 5) or the submeter-scale complexities observed in the subsurface architecture documented in the Margin unit, which are interpreted as Bs on Sol 1052 and mouth-bar deposits on Sol 1052 (Figs. 4 and 5), which cannot be easily explained by characteristics that define shoreline deposits or beach ridges. Multiple observations of prograding to aggrading geometries interpreted here as topsets and foresets are also challenging to reconcile with a shoreline depositional hypothesis over a Gilbert-style deltaic environment.

Outwash Delta

If the Margin unit was a glacial outwash plain (i.e., a sandur) where sediment-laden meltwater flowed from glacial ice sheet–wide floods, then we would expect a prograding depositional system that would be heterogeneous both in its overall geometry (varying lobe sizes) and in its structures (varying layers and clast classes) (*56*, *57*). However, the regular, repeated, consistent spacing between the laterally continuous reflectors and the presence of clinoform progradation favor a regular and continuous, subaqueous deposition in a "regular" deltaic environment over a sandur depositional environment (*58*, *59*). Therefore, the overall geometries observed, topset-foreset bedding, and fine-scale feature morphologies are most consistent with a subaqueous Gilbert-type deltaic formation, making it the most likely depositional environment for the Margin unit of Jezero crater. However, we cannot exclude the possibility that a glacial outwash delta could have also formed the fluvial and Gilbert deltaic features and structural complexity observed.

Implications

From the stratigraphic features mapped by RIMFAX, we can conclude that Jezero crater hosted an aqueous, possibly habitable environment capable of biosignature preservation, prior to the formation of Jezero's Western Delta. As predicted from orbital observations, RIMFAX confirms that the Margin unit is a distinct geologic unit from the Upper Fan, deposited earlier and distinct in composition as well as in physical area. This work suggests that there is some continuity of formation between the Margin unit and the Upper Fan, with a repeated process in Jezero crater but at distinctive different times of formation and deposition as well as likely different diagenetic processes that have preserved them. A body of water once fed Jezero crater and deposited sedimentary layers of varying scales, similar in size and morphology to those observed on the Western Fan (*16*, *20*). We suggest that this was once an extensive system that included the Margin unit, although it is now a buried remnant. However, the unit boundaries between the Margin unit and the Upper Fan are sharp with a distinct break in deltaic formation, and it is therefore unknown how much time elapsed between the formation of each unit. We speculate that the water body inside of the crater was laterally extensive and was moving in a south-southeast direction because the subsurface stratigraphic depositional and erosional layers dip in this general direction. While alternative deposition hypotheses were explored, the homogeneity of the radar facies and the presence of highly complex features found at multiple scales were not consistent with a fall, flow, surge, or lacustrine depositional mechanism (*11*), nor with igneous processes (whether intrusive or extrusive). These results are most consistent with an inferred history of fluvial regimes (*5*) and the onset of an earlier, broader deltaic architecture as others hypothesized from orbital observations. Overall, RIMFAX elucidates a broader fluvial system than what was observed from orbit, and indicates an extended window of fluvial deposition, aqueous alteration, and habitable conditions than previously envisioned at Jezero crater.

RIMFAX has revealed an earlier subsurface deltaic environment under the present-day delta, thereby extending the period of potential habitability for Jezero back further in time. This work also improves the chances of finding evidence for past life, as the formation of the present-day Delta Fan may have been rapid (*17*).

MATERIALS AND METHODS

Radar soundings were acquired with the GPR RIMFAX instrument using shallow and deep modes, as described (*18, 19, 22*). Unmigrated radargram profiles were processed and visualized as previously published (*18*, *19*). Single-sol and multi-sol 3D geologic profiles were generated in Python using the PyVista library (*60*). Multi-sol 3D

geologic profiles linked to HiRISE digital elevation models were generated in Julia (*61*) and displayed in the subsurface at 1× or 2× vertical exaggeration (see figure captions). Amplitude-normalized radiograms are blue and red colored, wherein high reflectivity values are indicated with red and low reflectivity values are indicated with blue. A propagation velocity of 0.126 m/ns at all depths is assumed, unless otherwise stated. All original data presented in this study can be found on the NASA Planetary Data System Geosciences Node, https://pds-geosciences.wustl.edu/missions/mars2020/rimfax.htm.

Supplementary Materials

This PDF file includes:

Supplementary Text

Figs. S1 and S2

REFERENCES

1. T. A. Goudge, J. F. Mustard, J. W. Head, C. I. Fassett, S. M. Wiseman, Assessing the mineralogy of the watershed and fan deposits of the Jezero crater paleolake system, Mars. *J. Geophys. Res. Planets* **120**, 775–808 (2015).
2. B. H. N. Horgan, R. B. Anderson, G. Dromart, E. S. Amador, M. S. Rice, The mineral diversity of Jezero crater: Evidence for possible lacustrine carbonates on Mars. *Icarus* **339**, 113526 (2020).
3. K. M. Stack, N. R. Williams, F. Calef, V. Z. Sun, K. H. Williford, K. A. Farley, S. Eide, D. Flannery, C. Hughes, S. R. Jacob, L. C. Kah, F. Meyen, A. Molina, C. Q. Nataf, M. Rice, P. Russell, E. Scheller, C. H. Seeger, W. J. Abbey, J. B. Adler, H. Amundsen, R. B. Anderson, S. M. Angel, G. Arana, J. Atkins, M. Barrington, T. Berger, R. Borden, B. Boring, A. Brown, B. L. Carrier, P. Conrad, H. Dypvik, S. A. Fagents, Z. E. Gallegos, B. Garczynski, K. Golder, F. Gomez, Y. Goreva, S. Gupta, S.-E. Hamran, T. Hicks, E. D. Hinterman, B. N. Horgan, J. Hurowitz, J. R. Johnson, J. Lasue, R. E. Kronyak, Y. Liu, J. M. Madariaga, N. Mangold, J. McClean, N. Miklusicak, D. Nunes, C. Rojas, K. Runyon, N. Schmitz, N. Scudder, E. Shaver, J. SooHoo, R. Spaulding, E. Stanish, L. K. Tamppari, M. M. Tice, N. Turenne, P. A. Willis, R. A. Yingst, Photogeologic map of the perseverance rover field site in Jezero crater constructed by the Mars 2020 science team. *Space Sci. Rev.* **216**, 127 (2020).
4. L. Mandon, C. Quantin-Nataf, P. Thollot, N. Mangold, L. Lozach, G. Dromart, P. Beck, E. Dehouck, S. Breton, C. Millot, M. Volat, Refining the age, emplacement and alteration scenarios of the olivine-rich unit in the Nili Fossae region, Mars. *Icarus* **336**, 113436 (2020).
5. N. Mangold, G. Dromart, V. Ansan, F. Salese, M. G. Kleinhans, M. Massé, C. Quantin-Nataf, K. M. Stack, Fluvial regimes, morphometry, and age of Jezero crater paleolake inlet valleys and their exobiological significance for the 2020 rover mission landing site. *Astrobiology* **20**, 994–1013 (2020).
6. T. A. Goudge, R. E. Milliken, J. W. Head, J. F. Mustard, C. I. Fassett, Sedimentological evidence for a deltaic origin of the western fan deposit in Jezero crater, Mars and implications for future exploration. *Earth Planet. Sci. Lett.* **458**, 357–365 (2017).
7. C. I. Fassett, J. W. Head, Fluvial sedimentary deposits on Mars: Ancient deltas in a crater lake in the Nili Fossae region. *Geophys. Res. Lett.* **32**, 2005GL023456 (2005).
8. S. Holm-Alwmark, K. M. Kinch, M. D. Hansen, S. Shahrzad, K. Svennevig, W. J. Abbey, R. B. Anderson, F. J. Calef, S. Gupta, E. Hauber, B. H. N. Horgan, L. C. Kah, J. Knade, N. B. Miklusicak, K. M. Stack, V. Z. Sun, J. D. Tarnas, C. Quantin-Nataf, Stratigraphic relationships in Jezero Crater, Mars: Constraints on the timing of fluvial-lacustrine activity from orbital observations. *J. Geophys. Res. Planets* **126**, e2021JE006840 (2021).
9. J. D. Tarnas, K. M. Stack, M. Parente, A. H. D. Koeppel, J. F. Mustard, K. R. Moore, B. H. N. Horgan, F. P. Seelos, E. A. Cloutis, P. B. Kelemen, D. Flannery, A. J. Brown, K. R. Frizzell, P. Pinet, Characteristics, origins, and biosignature preservation potential of carbonate-bearing rocks within and outside of Jezero Crater. *J. Geophys. Res. Planets* **126**, e2021JE006898 (2021).
10. A. M. Zastrow, T. D. Glotch, Distinct carbonate lithologies in Jezero Crater, Mars. *Geophys. Res. Lett.* **48**, e2020GL092365 (2021).
11. K. E. Cervantes-de La Cruz, E. Chacón-Baca, L. Montoya, "The Lake Alchichica from an astrobiological perspective" in *Lake Alchichica Limnology*, J. Alcocer, Ed. (Springer International Publishing, 2022), pp. 391–413; https://link.springer.com/10.1007/978-3-030-79096-7_23.
12. T. M. Burnie, I. M. Power, C. Paulo, H. Alçiçek, L. I. Falcón, Y. Lin, S. Wilson, Environmental and mineralogical controls on biosignature preservation in magnesium carbonate systems analogous to Jezero Crater, Mars. *Astrobiology* **23**, 513–535 (2023).
13. S. W. Ruff, V. E. Hamilton, A. D. Rogers, C. S. Edwards, B. H. N. Horgan, Olivine and carbonate-rich bedrock in Gusev crater and the Nili Fossae region of Mars may be altered ignimbrite deposits. *Icarus* **380**, 114974 (2022).
14. C. H. Kremer, J. F. Mustard, M. S. Bramble, A widespread olivine-rich ash deposit on Mars. *Geology* **47**, 677–681 (2019).
15. A. J. Brown, C. E. Viviano, T. A. Goudge, Olivine-carbonate mineralogy of the Jezero Crater region. *J. Geophys. Res. Planets* **125**, e2019JE006011 (2020).
16. S. C. Cuevas-Quiñones, J. J. Wray, F. Rivera-Hernández, J. B. Adler, Evidence for a composite volcano on the rim of Jezero crater on Mars. *Commun. Earth Environ.* **6**, 340 (2025).
17. J. R. Michalski, T. C. Onstott, S. J. Mojzsis, J. Mustard, Q. H. S. Chan, P. B. Niles, S. S. Johnson, The Martian subsurface as a potential window into the origin of life. *Nat. Geosci.* **11**, 21–26 (2018).
18. S.-E. Hamran, D. A. Paige, A. Allwood, H. E. F. Amundsen, T. Berger, S. Brovoll, L. Carter, T. M. Casademont, L. Damsgård, H. Dypvik, S. Eide, A. G. Fairén, R. Ghent, J. Kohler, M. T. Mellon, D. C. Nunes, D. Plettemeier, P. Russell, M. Siegler, M. J. Øyan, Ground penetrating radar observations of subsurface structures in the floor of Jezero crater, Mars. *Sci. Adv.* **8**, eabp8564 (2022).
19. D. A. Paige, S.-E. Hamran, H. E. F. Amundsen, T. Berger, P. Russell, R. Kakaria, M. T. Mellon, S. Eide, L. M. Carter, T. M. Casademont, D. C. Nunes, E. S. Shoemaker, D. Plettemeier, H. Dypvik, S. Holm-Alwmark, B. H. N. Horgan, Ground penetrating radar observations of the contact between the western delta and the crater floor of Jezero crater, Mars. *Sci. Adv.* **10**, eadi8339 (2024).
20. E. S. Shoemaker, T. M. Casademont, L. M. Carter, P. Russell, H. Dypvik, S. Alwmark, B. H. N. Horgan, H. E. F. Amundsen, S. Eide, S.-E. Hamran, D. A. Paige, S. Gupta, E. L. Cardarelli, U. Árting, T. Berger, S. Brovoll, Observations of igneous subsurface stratigraphy during the Jezero crater floor rapid traverse from the RIMFAX ground-penetrating radar. *Planet. Sci. J.* **5**, 191 (2024).
21. K. M. Stack, L. R. W. Ives, S. Gupta, M. P. Lamb, M. Tebolt, G. Caravaca, J. P. Grotzinger, P. Russell, D. L. Shuster, A. J. Williams, H. Amundsen, S. Alwmark, A. M. Annex, R. Barnes, J. Bell, O. Beyssac, T. Bosak, L. S. Crumpler, E. Dehouck, S. J. Gwizd, K. Hickman-Lewis, B. H. N. Horgan, J. Hurowitz, H. Kalucha, O. Kanine, C. Lesh, J. Maki, N. Mangold, N. Randazzo, C. Seeger, R. M. E. Williams, A. Brown, E. Cardarelli, H. Dypvik, D. Flannery, J. Frydenvang, S.-E. Hamran, J. I. Núñez, D. Paige, J. I. Simon, M. Tice, C. Tate, R. C. Wiens, Sedimentology and stratigraphy of the Shenandoah formation, western fan, Jezero crater, Mars. *J. Geophys. Res. Planets* **129**, e2023JE008187 (2024).
22. S.-E. Hamran, D. A. Paige, H. E. F. Amundsen, T. Berger, S. Brovoll, L. Carter, L. Damsgård, H. Dypvik, J. Eide, S. Eide, R. Ghent, Ø. Helleren, J. Kohler, M. Mellon, D. C. Nunes, D. Plettemeier, K. Rowe, P. Russell, M. J. Øyan, Radar imager for Mars' subsurface experiment—RIMFAX. *Space Sci. Rev.* **216**, 128 (2020).
23. S. Arcone, S. Campbell, W. T. Pfeffer, GPR profiles of glacial till and its transition to bedrock: Interpretation of water content, depth and signal loss from diffractions. *J. Environ. Eng. Geophys.* **19**, 207–228 (2014).
24. W. Guo, C. Dong, C. Lin, T. Zhang, Z. Zhao, J. Li, 3D sedimentary architecture of sandy braided river, based on outcrop, unmanned aerial vehicle and ground penetrating radar data. *Minerals* **12**, 739 (2022).
25. N. Mangold, G. Caravaca, S. Gupta, R. M. E. Williams, G. Dromart, O. Gasnault, S. Le Mouélic, G. Paar, J. Bell, O. Beyssac, N. Carlot, A. Cousin, E. Dehouck, B. Horgan, L. C. Kah, J. Lasue, S. Maurice, J. I. Núñez, D. Shuster, K. M. Stack, B. P. Weiss, R. C. Wiens, Architecture of fluvial and deltaic deposits exposed along the eastern edge of the western fan of Jezero crater, Mars. *J. Geophys. Res. Planets* **129**, e2023JE008204 (2024).
26. N. Mangold, S. Gupta, O. Gasnault, G. Dromart, J. D. Tarnas, S. F. Sholes, B. Horgan, C. Quantin-Nataf, A. J. Brown, S. Le Mouélic, R. A. Yingst, J. F. Bell, O. Beyssac, T. Bosak, F. Calef, B. L. Ehlmann, K. A. Farley, J. P. Grotzinger, K. Hickman-Lewis, S. Holm-Alwmark, L. C. Kah, J. Martinez-Frias, S. M. McLennan, S. Maurice, J. I. Nuñez, A. M. Ollila, P. Pilleri, J. W. Rice, M. Rice, J. I. Simon, D. L. Shuster, K. M. Stack, V. Z. Sun, A. H. Treiman, B. P. Weiss, R. C. Wiens, A. J. Williams, N. R. Williams, K. H. Williford, Perseverance rover reveals an ancient delta-lake system and flood deposits at Jezero crater, Mars. *Science* **374**, 711–717 (2021).
27. G. K. Gilbert, The topographic features of lake shores. *5th Annu. Rep. U. S. Geol. Surv.*, 69–123 (1885).
28. S. Leleu, A. J. Hartley, B. P. J. Williams, Large-scale alluvial architecture and correlation in a Triassic pebbly braided river system, lower Wolfville Formation (Fundy Basin, Nova Scotia, Canada). *J. Sediment. Res.* **79**, 265–286 (2009).
29. D. Franke, J. Hornung, M. Hinderer, A combined study of radar facies, lithofacies and three-dimensional architecture of an alpine alluvial fan (Illgraben fan, Switzerland). *Sedimentology* **62**, 57–86 (2015).
30. J. Vandenberghe, R. A. Van Overmeeren, Ground penetrating radar images of selected fluvial deposits in the Netherlands. *Sediment. Geol.* **128**, 245–270 (1999).
31. G. Caravaca, G. Dromart, N. Mangold, S. Gupta, L. C. Kah, C. Tate, R. M. E. Williams, S. Le Mouélic, O. Gasnault, J. Bell, O. Beyssac, J. I. Nuñez, N. Randazzo, J. Rice, L. S. Crumpler, A. Williams, P. Russel, K. M. Stack, K. A. Farley, S. Maurice, R. C. Wiens, Depositional facies and sequence stratigraphy of Kodiak Butte, Western Delta of Jezero Crater, Mars. *J. Geophys. Res. Planets* **129**, e2023JE008205 (2024).
32. J. Li, H. Liu, X. Meng, D. Duan, H. Lu, J. Zhang, F. Zhang, D. Elsworth, B. T. Cardenas, M. Manga, B. Zhou, G. Fang, Ancient ocean coastal deposits imaged on Mars. *Proc. Natl. Acad. Sci. U.S.A.* **122**, e2422213122 (2025).
33. A. S. McEwen, E. M. Eliason, J. W. Bergstrom, N. T. Bridges, C. J. Hansen, W. A. Delamere, J. A. Grant, V. C. Gulick, K. E. Herkenhoff, L. Keszthelyi, R. L. Kirk, M. T. Mellon, S. W. Squyres,

N. Thomas, C. M. Weitz, Mars Reconnaissance Orbiter's High Resolution Imaging Science Experiment (HiRISE). *J. Geophys. Res. Planets* **112**, 2005JE002605 (2007).

34. A. M. Morgan, A. D. Howard, D. E. J. Hobley, J. M. Moore, W. E. Dietrich, R. M. E. Williams, D. M. Burr, J. A. Grant, S. A. Wilson, Y. Matsubara, Sedimentology and climatic environment of alluvial fans in the martian Saheki crater and a comparison with terrestrial fans in the Atacama Desert. *Icarus* **229**, 131–156 (2014).
35. S. L. Murchie, J. F. Mustard, B. L. Ehlmann, R. E. Milliken, J. L. Bishop, N. K. McKeown, E. Z. Noe Dobrea, F. P. Seelos, D. L. Buczkowski, S. M. Wiseman, R. E. Arvidson, J. J. Wray, G. Swayze, R. N. Clark, D. J. Des Marais, A. S. McEwen, J. Bibring, A synthesis of Martian aqueous mineralogy after 1 Mars year of observations from the Mars Reconnaissance Orbiter. *J. Geophys. Res. Planets* **114**, 2009JE003342 (2009).
36. J. G. McPherson, G. Shanmugam, R. J. Moiola, Fan-deltas and braid deltas: Varieties of coarse-grained deltas. *Geol. Soc. Am. Bull.* **99**, 331 (1987).
37. A. Fontana, P. Mozzi, M. Marchetti, Alluvial fans and megafans along the southern side of the Alps. *Sediment. Geol.* **301**, 150–171 (2014).
38. A. M. Harvey, Alluvial fan dissection: Relationships between morphology and sedimentation. *Geol. Soc. Lond. Spec. Publ.* **35**, 87–103 (1987).
39. D. Bowman, "Fan entrenchment" in *Principles of Alluvial Fan Morphology* (Springer, 2019), pp. 61–67; https://link.springer.com/10.1007/978-94-024-1558-2_8.
40. S. Y. J. Lai, F. Wu, Two-stage transition from Gilbert to hyperpycnal delta in reservoir. *Geophys. Res. Lett.* **48**, e2021GL093661 (2021).
41. A. M. A. Zarza, V. P. Wright, J. P. Calvo, M. A. G. Del Cura, Soil-landscape and climatic relationships in the middle Miocene of the Madrid Basin. *Sedimentology* **39**, 17–35 (1992).
42. E. L. Cardarelli, J. R. Bargar, C. A. Francis, Diverse *Thaumarchaeota* dominate subsurface ammonia-oxidizing communities in semi-arid floodplains in the Western United States. *Microb. Ecol.* **80**, 778–792 (2020).
43. L. Reji, E. L. Cardarelli, K. Boye, J. R. Bargar, C. A. Francis, Diverse ecophysiological adaptations of subsurface *Thaumarchaeota* in floodplain sediments revealed through genome-resolved metagenomics. *ISME J.* **16**, 1140–1152 (2022).
44. B. L. Ehlmann, J. F. Mustard, S. L. Murchie, F. Poulet, J. L. Bishop, A. J. Brown, W. M. Calvin, R. N. Clark, D. J. D. Marais, R. E. Milliken, L. H. Roach, T. L. Roush, G. A. Swayze, J. J. Wray, Orbital identification of carbonate-bearing rocks on Mars. *Science* **322**, 1828–1832 (2008).
45. J. S. Scoates, R. F. J. Scoates, The Muskox intrusion: Overview of a major open-system layered intrusion and its role as a sub-volcanic magma reservoir in the Mackenzie large igneous province. *Lithos* **474-475**, 107560 (2024).
46. T. Irvine, "Sedimentary structures in igneous intrusions with particular reference to the Duke Island ultramafic complex" in *Primary Sedimentary Structures and Their Hydrodynamic Interpretation* (SEPM Society for Sedimentary Geology, 1965).
47. Y. Liu, M. M. Tice, M. E. Schmidt, A. H. Treiman, T. V. Kizovski, J. A. Hurowitz, A. C. Allwood, J. Henneke, D. A. K. Pedersen, S. J. VanBommel, M. W. M. Jones, A. L. Knight, B. J. Orenstein, B. C. Clark, W. T. Elam, C. M. Heirwegh, T. Barber, L. W. Beegle, K. Benzerara, S. Bernard, O. Beyssac, T. Bosak, A. J. Brown, E. L. Cardarelli, D. C. Catling, J. R. Christian, E. A. Cloutis, B. A. Cohen, S. Davidoff, A. G. Fairén, K. A. Farley, D. T. Flannery, A. Galvin, J. P. Grotzinger, S. Gupta, J. Hall, C. D. K. Herd, K. Hickman-Lewis, R. P. Hodyss, B. H. N. Horgan, J. R. Johnson, J. L. Jørgensen, L. C. Kah, J. N. Maki, L. Mandon, N. Mangold, F. M. McCubbin, S. M. McLennan, K. Moore, M. Nachon, P. Nemere, L. D. Nothdurft, J. I. Núñez, L. O'Neil, C. M. Quantin-Nataf, V. Sautter, D. L. Shuster, K. L. Siebach, J. I. Simon, K. P. Sinclair, K. M. Stack, A. Steele, J. D. Tarnas, N. J. Tosca, K. Uckert, A. Udry, L. A. Wade, B. P. Weiss, R. C. Wiens, K. H. Williford, M.-P. Zorzano, An olivine cumulate outcrop on the floor of Jezero crater, Mars. *Science* **377**, 1513–1519 (2022).
48. E. C. Ferré, S. M. Maes, K. C. Butak, The magnetic stratification of layered mafic intrusions: Natural examples and numerical models. *Lithos* **111**, 83–94 (2009).
49. R. M. Latypov, O. Namur, Y. Bai, S. J. Barnes, S. Chistyakova, M. B. Holness, G. Iacono-Marziano, W. A. J. Kruger, B. O'Driscoll, W. D. Smith, V. J. Virtanen, C. Y. Wang, C.-M. Xing, B. Charlier, Layered intrusions: Fundamentals, novel observations and concepts, and controversial issues. *Earth Sci. Rev.* **249**, 104653 (2024).
50. W. D. Smith, M. C. Jenkins, C. T. Augustin, V. J. Virtanen, Z. Vukmanovic, B. O'Driscoll, Layered intrusions in the Precambrian: Observations and perspectives. *Precambrian Res.* **415**, 107615 (2024).
51. B. L. Ehlmann, J. F. Mustard, G. A. Swayze, R. N. Clark, J. L. Bishop, F. Poulet, D. J. Des Marais, L. H. Roach, R. E. Milliken, J. J. Wray, O. Barnouin-Jha, S. L. Murchie, Identification of hydrated silicate minerals on Mars using MRO-CRISM: Geologic context near Nili Fossae and implications for aqueous alteration. *J. Geophys. Res. Planets* **114**, 2009JE003339 (2009).
52. O. Beyssac, O. Forni, A. Cousin, A. Udry, L. C. Kah, L. Mandon, O. E. Clavé, Y. Liu, F. Poulet, C. Quantin Nataf, O. Gasnault, J. R. Johnson, K. Benzerara, P. Beck, E. Dehouck, N. Mangold, C. Alvarez Llamas, R. B. Anderson, G. Arana, R. Barnes, S. Bernard, T. Bosak, A. J. Brown, K. Castro, B. Chide, S. M. Clegg, E. Cloutis, T. Fouchet, T. Gabriel, S. Gupta, G. Lacombe, J. Lasue, S. Le Mouelic, G. Lopez-Reyes, J. M. Madariaga, F. M. McCubbin, S. M. McLennan, J. A. Manrique, P. Y. Meslin, F. Montmessin, J. Núñez, A. M. Ollila, A. Ostwald, P. Pilleri, P. Pinet, C. Royer, S. K. Sharma, S. Schröder, J. I. Simon, M. J. Toplis, M. Veneranda, P. A. Willis, S. Maurice, R. C. Wiens, The SuperCam Team, Petrological traverse of the olivine cumulate Séítah formation at Jezero crater, Mars: A perspective from SuperCam onboard perseverance. *J. Geophys. Res. Planets* **128**, e2022JE007638 (2023).
53. E. L. Scheller, J. R. Hollis, E. L. Cardarelli, A. Steele, L. W. Beegle, R. Bhartia, P. Conrad, K. Uckert, S. Sharma, B. L. Ehlmann, W. J. Abbey, S. A. Asher, K. C. Benison, E. L. Berger, O. Beyssac, B. L. Bleefeld, T. Bosak, A. J. Brown, A. S. Burton, S. V. Bykov, E. Cloutis, A. G. Fairén, L. DeFlores, K. A. Farley, D. M. Fey, T. Fornaro, A. C. Fox, M. Fries, K. Hickman-Lewis, W. F. Hug, J. E. Huggett, S. Imbeah, R. S. Jakubek, L. C. Kah, P. Kelemen, M. R. Kennedy, T. Kizovski, C. Lee, Y. Liu, L. Mandon, F. M. McCubbin, K. R. Moore, B. E. Nixon, J. I. Núñez, C. R. Sanchez-Vahamonde, R. D. Roppel, M. Schulte, M. A. Sephton, S. K. Sharma, S. Siljeström, S. Shkolyar, D. L. Shuster, J. I. Simon, R. J. Smith, K. M. Stack, K. Steadman, B. P. Weiss, A. Werynski, A. J. Williams, R. C. Wiens, K. H. Williford, K. Winchell, B. Wogsland, A. Yanchilina, R. Yingling, M.-P. Zorzano, Aqueous alteration processes in Jezero crater, Mars—Implications for organic geochemistry. *Science* **378**, 1105–1110 (2022).
54. D. E. Wilkins, W. P. Clement, "Paleolake shoreline sequencing using ground penetrating radar: Lake Alvord, Oregon, and Nevada" in *Special Paper 432: Stratigraphic Analyses Using GPR* (Geological Society of America, 2007), pp. 103–110; https://pubs.geoscienceworld.org/books/book/586/chapter/3804030/.
55. A. Neal, N. I. Pontee, K. Pye, J. Richards, Internal structure of mixed-sand-and-gravel beach deposits revealed using ground-penetrating radar. *Sedimentology* **49**, 789–804 (2002).
56. D. Harrison, N. Ross, A. J. Russell, S. J. Jones, Ground-penetrating radar (GPR) investigations of a large-scale buried ice-marginal landsystem, Skeiðarársandur, SE Iceland. *Boreas* **51**, 824–846 (2022).
57. P. M. Marren, Magnitude and frequency in proglacial rivers: A geomorphological and sedimentological perspective. *Earth Sci. Rev.* **70**, 203–251 (2005).
58. S. Patruno, W. Helland-Hansen, Clinoforms and clinoform systems: Review and dynamic classification scheme for shorelines, subaqueous deltas, shelf edges and continental margins. *Earth Sci. Rev.* **185**, 202–233 (2018).
59. R. Rubi, S. Rohais, S. Bourquin, I. Moretti, G. Desaubliaux, Processes and typology in Gilbert-type delta bottomset deposits based on outcrop examples in the Corinth Rift. *Mar. Pet. Geol.* **92**, 193–212 (2018).
60. C. Sullivan, A. Kaszynski, PyVista: 3D plotting and mesh analysis through a streamlined interface for the Visualization Toolkit (VTK). *J. Open Source Softw.* **4**, 1450 (2019).
61. J. Bezanson, A. Edelman, S. Karpinski, V. B. Shah, Julia: A fresh approach to numerical computing. *SIAM Rev.* **59**, 65–98 (2017).

Acknowledgments: The development of the RIMFAX instrument was supported by the Norwegian Space Agency and the Norwegian Defense Research Establishment (FFI). We thank the Mars 2020 Engineering and Operations Teams for support in acquiring these data and observations. We also thank the reviewers for valuable feedback and suggestions, which improved this manuscript. **Funding:** This work was supported by Jet Propulsion Laboratory, through NASA's Mars Exploration Program via subcontracts from the California Institute of Technology/Jet Propulsion Laboratory (subcontract 1512986) (to D.A.P., E.L.C, P.R., and B.H.); Research Council of Norway, grant no. 309835 (to S.-E.H. and T.B.); and Centre for Space Sensors and Systems (CENSSS), through their SFI Centre for Research-Based Innovation program (to S.-E.H. and T.B.). Part of this research was carried out at the Jet Propulsion Laboratory, California Institute of Technology, under a contract with the National Aeronautics and Space Administration (80NM0018D0004). **Author contributions:** Conceptualization: E.L.C., U.A., S.-E.H., D.A.P., F.A., and A.B. Methodology: E.L.C., S.-E.H., P.R., F.A., and D.A.P. Investigation: E.L.C., D.A.P., S.B., T.B., B.H., and U.A. Formal analysis: E.L.C., F.A., S.-E.H., and T.B. Software: E.L.C., F.A., S.-E.H., S.B., and T.B. Data curation: D.A.P., F.A., S.B., and T.B. Validation: E.L.C., U.A., and G.C. Visualization: E.L.C., S.-E.H., D.A.P., F.A., T.B., P.R., U.A., G.C., and A.B. Resources: S.-E.H., D.A.P., and S.B. Writing—original draft: E.L.C. and U.A. Writing—review and editing: E.L.C., S.-E.H., D.A.P., P.R., U.A., A.B., B.H., G.C., and F.A. Supervision: D.A.P. Project administration: E.L.C., D.A.P., and S.-E.H. Funding acquisition: D.A.P. and S.-E.H. **Competing interests:** The authors declare that they have no competing interests. **Data, code, and materials availability:** All data and code needed to evaluate and reproduce the results in the paper are present in the paper and/or the Supplementary Materials. All original data presented in this study for the purposes of reproducing or extending the analyses can be found on the NASA Planetary Data System Geosciences Node, https://pds-geosciences.wustl.edu/missions/mars2020/rimfax.htm. This study did not generate new materials.

Submitted 8 June 2025
Accepted 19 February 2026
Published 18 March 2026
10.1126/sciadv.adz6095
