# Supplementary material for: Ground penetrating radar observations of ancient large-scale deltaic structures in Jezero crater, Mars
Source: Sci Adv. 2026 Mar 18;12(12):eadz6095. doi: 10.1126/sciadv.adz6095 (PMC12998504; doi:10.1126/sciadv.adz6095)
Supplement: Supplementary file 2 — Supplementary Text Figs. S1 and S2 [file sciadv.adz6095_sm.pdf]

Science Advances
AAAS

Supplementary Materials for

Ground penetrating radar observations of ancient large-scale deltaic structures in Jezero crater, Mars

Emily L. Cardarelli *et al.*

Corresponding author: Emily L. Cardarelli, ecardare@epss.ucla.edu

Sci. Adv. **12**, eadz6095 (2026)
DOI: 10.1126/sciadv.adz6095

This PDF file includes:

Supplementary Text
Figs. S1 and S2

Supplementary Text

Topography and extent of the Margin Unit

Understanding the topographic as well as paleotopographic changes experienced over the campaign are a key component to the elevation, slope, aspect, and landform features present today and may have influenced the stratigraphic features observed on the surface or preserved in the subsurface. For example, the steeper elevational changes are observed in the most northwestern and far western portion of the Margin Unit may have influenced the preservation of riverine/deltaic features and supported active erosional episodes in the past.

We entered the Margin Unit on the Sol 909 traverse at ~ -2415 m elevation after descending ~5 m across the channel at the base of the Margin Unit to the Hans Amundsen Memorial Workspace (end of drive location, Sol 909), which represents the lowest point explored in the Margin Unit. We drove on parallel to the basal channel of the Margin Unit. There was a consistent elevation (-2410 m) until turning west into the unit's interior (Sol 927, Bernier Island). There was a gradual increase in topography from Bernier Island to the entrance of the Gnaraloo Bay area (-2400 m elevation). The elevation of the northern basal channel is roughly 20 m higher than at the entrance of the Margin Unit, crossed in the Sol 909 traverse. We also report a local low point in the area where we transition back onto bedrock (Sol 1002) and begin the Margin Unit western traverse (Sol 1002-1159). From Sols 1002-1041, the traverse elevation remains relatively consistent (-2410 m) and from Sols 1041-1045 the elevation is roughly 10 m higher (-2400 m). A 10 m change in elevation is observed over the Sol 1046 traverse (200 m, -2390 m). We also observed a 5 m change in elevation over the ~100 m traverse (Sol 1047) as well as from Sol 1048 to Bunsen Peak (-2385 m to -2380 m) and from Bunsen Peak to Sol 1101 (-2380 m to -2375 m). However, a 15 m elevational change is observable over a ~515 m traverse distance over Sols 1101 to 1120. Over the Sol 1129-1159 traverses, there is a 11 m elevational change from -2355 m to -2364 m - with the base of the Margin Unit detected at 3 m below the surface at the sol 1159 end of drive location as well as at 36 m belowground (at -2351 m elevation) at roughly 230 m into the traverse at Sol 1140.

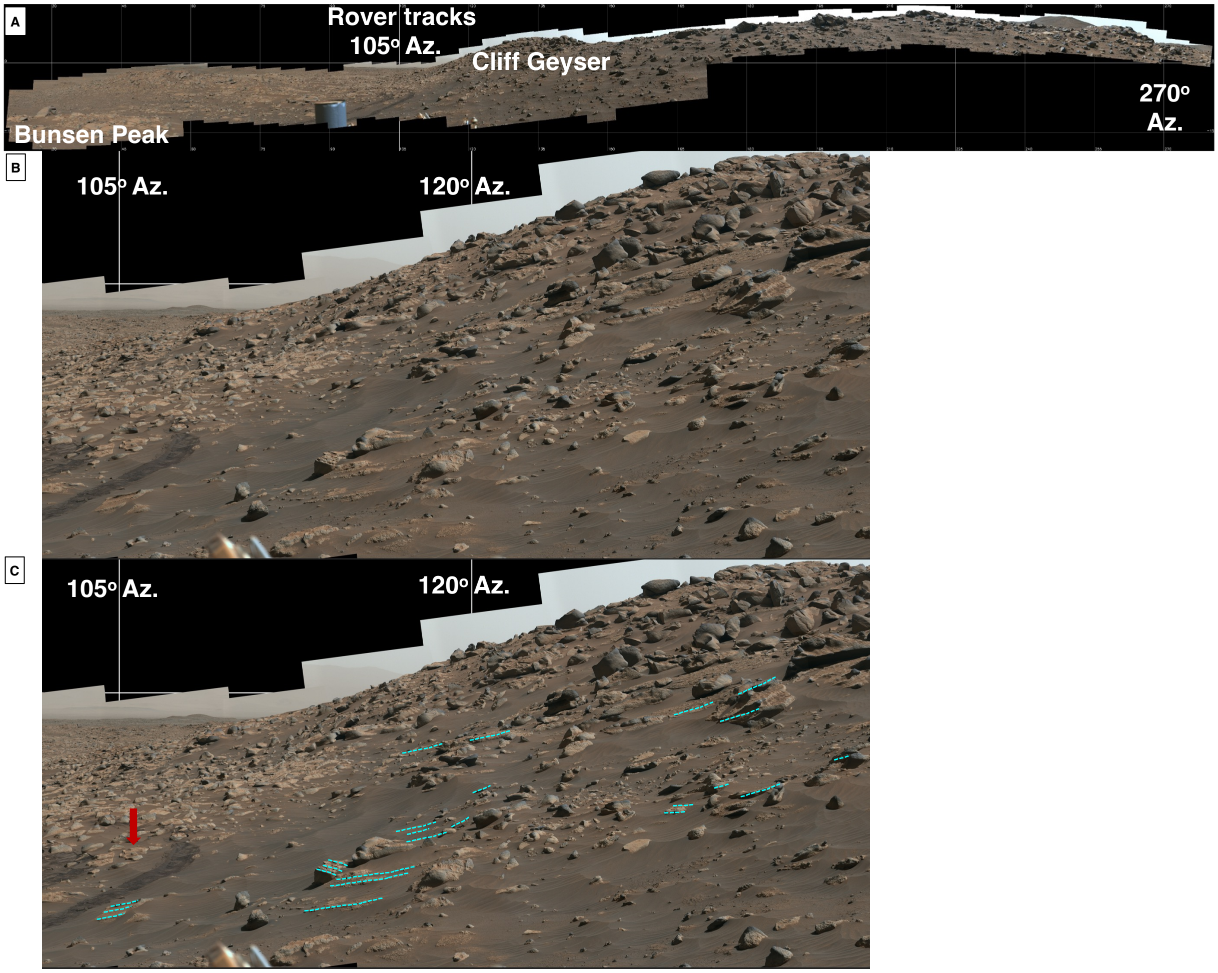

Fig. S1. Surface expressions of subsurface dipping reflectors detected on the Sol 1052 traverse viewed from the end of drive location looking behind *Perseverance.* Mastcam-Z mosaic QZCAM_SOL1054_ZCAM09075_R0_Z110_CLIFF_ GEYSER_MARGIN_UNIT_E01 wherein the subsurface layers observed by RIMFAX (Sol 1045-1055 traverses) are dipping towards the basin outcrops of them are easier to see when looking behind the rover. 105° azimuth viewing angle from the end of drive location shows the point in the rover tracks where Perseverance turned from a westerly drive heading to the northwest **(A, B, C - red arrow)**. Cliff Geyser appears to be pervasively layered and those layers appear to sweep down to the location of the rover traverse (red arrow), the projected area wherein RIMFAX reflectors come to the surface (C - cyan traces).

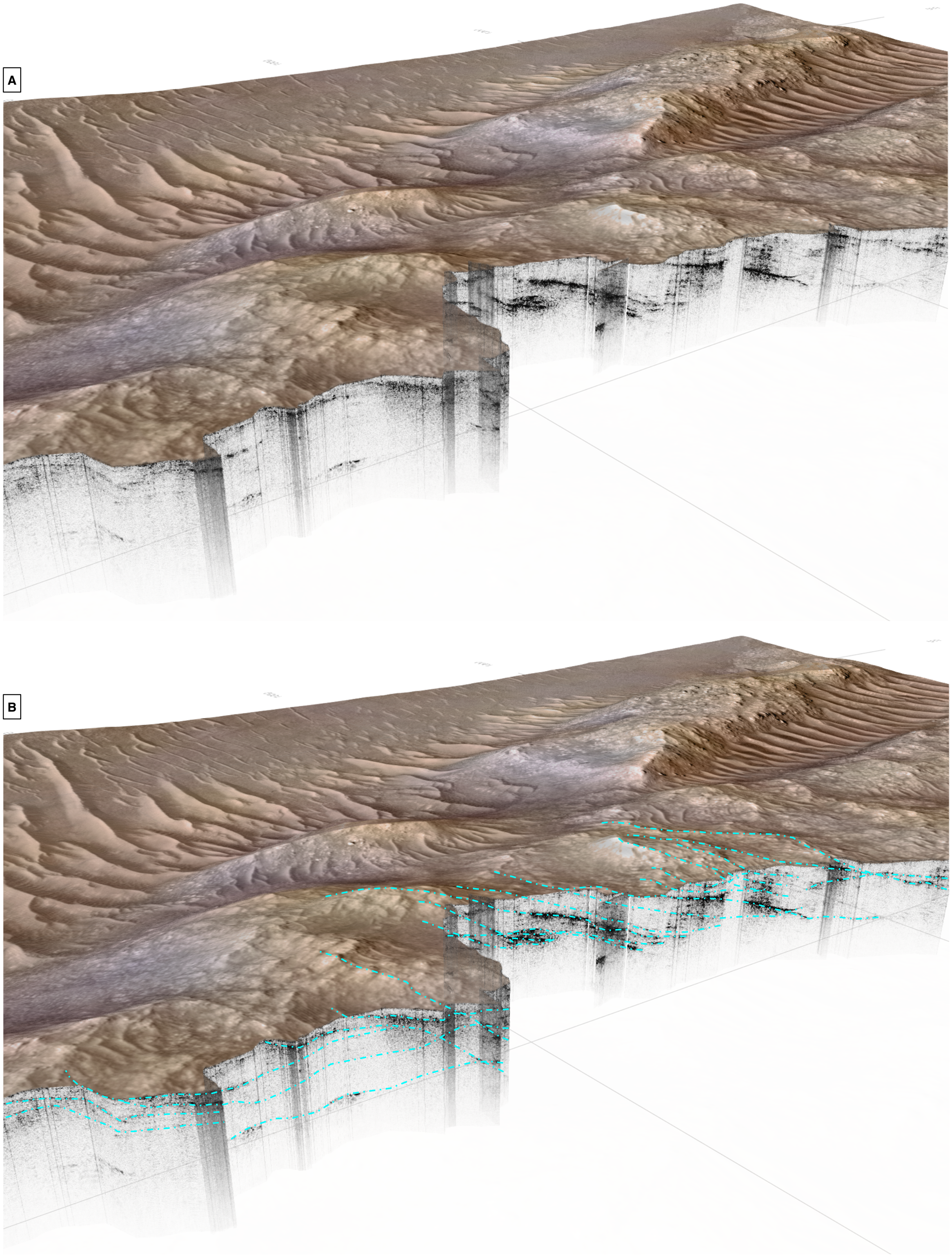
A
B

Fig. S2. Cross-sectional projected 3D views of the RIMFAX reflection amplitude radargrams of the Margin Unit acquired over Sol 1052. (A) In the Margin Unit, strongly reflecting layers that are dark in appearance and weakly reflecting lithologies appear as light. The projected radargram is shown with the HiRISE digital elevation model data and layers are traced (cyan dotted lines) from the subsurface to corresponding surficial topographic features **(B)**.
